# Spices Beyond Antioxidants: From the Gut to the Brain

**DOI:** 10.1093/nutrit/nuaf176

**Published:** 2026-05-26

**Authors:** Tatiana Diacova, David Heber, Zhaoping Li

**Affiliations:** Department of Medicine, Center for Human Nutrition, David Geffen School of Medicine, Los Angeles, CA, 90095, United States; Department of Medicine, Center for Human Nutrition, David Geffen School of Medicine, Los Angeles, CA, 90095, United States; Department of Medicine, Center for Human Nutrition, David Geffen School of Medicine, Los Angeles, CA, 90095, United States

**Keywords:** spices, bioactives, (poly)phenols, microbiome, human health

## Abstract

**Objectives:**

The objective of this review was to summarize evidence of the effects of select spices/herbs on human health with a focus on the work conducted at the University of California–Los Angeles (UCLA) Center for Human Nutrition.

**Background:**

Herbs/spices have been used in various countries around the world for centuries. The purposes for using herbs/spices include improvement in food organoleptic properties and use as food preservatives and medicine. As interest in ethnic cuisines is gaining popularity, more Americans are interested in adding spices/herbs to their daily diets for health benefits. Health benefits conferred by herbs/spices include protection against oxidative stress, neurodegeneration, cardiovascular disease among others and may be at least partially due to their high (poly)phenolic content. (Poly)phenols are not accessible by the human digestive enzymes and are metabolized by the gut microbiome, earning them the status of “prebiotics.” This is confirmed by a vast body of evidence pointing to the herbs’/spices’ ability to affect gut microbiota composition/functionality.

**Methods:**

In vitro experiments and human trials conducted at the UCLA Center for Human Nutrition were collated and results summarized. Reference lists of these publications were scanned and relevant literature extracted. Multiple additional searches relating to the select herbs/spices and their effects on human health were conducted in PubMed.

**Results:**

A total of 8 human trials and 12 in vitro experiments were conducted at the UCLA Center for Human Nutrition between 2010 and 2024. These experiments included interventions with individual herbs/spices, including cinnamon, chili pepper, and turmeric, as well as spice mixes. Additional relevant original research and reviews/meta-analyses were identified and included to supplement the discussions.

**Conclusion:**

While further research of herbs/spices is undeniably warranted, several considerations should be kept in mind. These include the relatively small amounts of herbs/spices consumed by the general population on a daily basis, cooking methods, as well as effects of digestive/metabolic processes on the bioavailability of herb/spice (poly)phenols. While much is already known, there are still substantial gaps in knowledge that need to be addressed.

## INTRODUCTION

Herbs and spices have been used in various countries around the world for centuries. Most of the known spices originate from the Mediterranean countries, including the Middle East or Asia, and many have been used since ancient Egyptian and Roman times.^1^ Some of the major uses of spices and herbs include addition to food for improvement in flavor and organoleptic properties, as well as usage as a preservative and medicine.^1^

National dietary guidelines from several countries (eg, the United States, United Kingdom, and Australia) recommend the consumption of spices to lower sodium intake.^2–4^ The National Institutes of Health (NIH)–funded Dietary Approaches to Stop Hypertension (DASH) healthy eating plan replaces salt with spices and herbs as a means to lower blood pressure without medication. A recent study by Li et al.^5^ showed that individuals with a high preference for spices had a lower salt intake and blood pressure compared with individuals who disliked spicy foods. It was also demonstrated that salt intake was associated with regional metabolic activity in the insula and orbitofrontal cortex (OFC) areas of the brain.^5^ The administration of capsaicin (chili pepper) in the Li et al. study enhanced metabolic activity in the insula and OFC regions of the brain in response to high-salt stimuli, counteracting the salt-induced differences in regional brain metabolism.^5^ These findings suggest that the consumption of spicy foods may be able to reduce salt preference, daily salt intake, and blood pressure by modifying the neural processing of salty tastes in the brain.^5^ Besides blood pressure–lowering activity, the addition of herbs and spices to one’s diet may offer benefits for the prevention and management of several additional age-related chronic diseases, including cardiovascular and neurodegenerative diseases, chronic inflammation, cancer, obesity, and type 2 diabetes.^6–18^

A very large observational study in China correlated the daily consumption of spices with reduced disease risk and causes of death in 487 375 individuals between the ages of 30 and 79 years.^19^ The overall conclusion was that participants who added spices to their foods almost every day had a 14% lower risk of death compared with those who consumed spicy foods less than once per week.^19^ It was also suggested that frequent consumption of spicy foods was linked to a lower risk of death from cancer, ischemic heart diseases, and respiratory diseases.^19^

The increasing recognition of the health benefits of herbs and spices is driving exponential growth of sales and consumption around the world, with an estimated 2024 revenue of $172 billion globally.^20^ On both a volume and value basis, the United States is the world’s largest spice importer and consumer, with both imports and consumption trending up for the past 10 years.^21^ As the interest in ethnic cuisines is gaining popularity, more and more Americans are interested in adding spices and herbs to their daily diets. In fact, a recent cross-sectional study of 703 adults in the midwestern United States explored perceptions and knowledge about the use of spices for health promotion.^22^ The results indicated that nearly 50% of the participants were interested in learning about health benefits of spices and were willing to use spices for their health benefits.^22^ The majority of the participants were currently using one or more spices on a daily basis and believed that ginger, garlic, and cinnamon could promote good health and wellness.^22^

### Chemical Composition of Spices/Herbs and Their Biological Activity

The difference between spices and herbs is in the part of the plant they come from.^23^ Herbs usually come from fresh leaves and flowers, while spices are derived from the root, seeds, bark, stem, berries, or buds and are usually dried.^23^ Spices and herbs can be further classified into various groups based on their flavor/taste, taxonomy, and the specific part of the plant they originated from (see Table 1).^23^ Herbs have a more delicate, fresh, and green flavor, and are often added toward the end of cooking or used fresh in salads, sauces, and garnishes. Spices tend to have stronger concentrated flavors and are used during cooking as well as afterwards in marinades, rubs, and slow-cooked dishes to enhance flavor.^23^

**Table 1.** Phenolic Composition of Commonly Used Herbs/Spices

	Type	Plant part	Phenolic composition	Total phenolic content, mg GAE/g	References
Spices and herbs					
Cloves	Aromatic spice	Flower/bud, fruits/berries	Eugenol, isoeugenol, acetyleugenol, sesquiterpene, pinene, vanillin, gallic acid, flavonoids, phenolic acids	232.99 (ground bud)	^24^ ^,^ ^25^
Oregano	Aromatic herb	Leaves	Apigenin, quercetin, luteolin, myricetin, diosmetin, eriodictyol, carvacrol, thymol, rosmarinic, caffeic, p-coumaric, protocatechuic acid	67.03 (dried leaf)	^24^ ^,^ ^26^
Thyme	Aromatic herb	Leaves	Phenolic acids (gallic acid, caffeic acid, rosmarinic acid), thymol, phenolic diterpenes, flavonoids	59.48 (dried leaf)	^25^ ^,^ ^26^
Cinnamon	Aromatic spice	Bark	Eugenol, limonene, terpineol, catechins, proanthocyanidins, tannins, linalool, safrole, pinene, methyleugenol, benzaldehyde	42.90 (dried ground quill)	^26^ ^,^ ^27^
Parsley	Aromatic herb	Leaves	Apigenin, luteolin, kaempferol, myricetin, quercetin, caffeic acid	24.33 (dried leaf)	^25^
Black pepper	Hot spice	Fruits/berries	Piperine, pinene, camphene, limonene, terpenes, piperidine, isoquercetin, sarmentine	16.69 (ground peppercorn)	^24^ ^,^ ^25^
Paprika	Mild flavor spice	Fruits	Gallic acid, chlorogenic acid, caffeic acid, p-coumaric acid, ferulic acid, quercetin, myricetin, kaempferol, xanthophylles	15.16 (dried ground fruit)	^25^ ^,^ ^28^
Rosemary	Aromatic herb	Leaves and twigs	Carnosol, rosmanol, geraniol, pinene, limonene, apigenin, naringin, luteolin, rosmarinic, vanillic, ursolic, caffeic acids	14.62 (dried leaf)	^25^ ^,^ ^26^
Ginger	Hot/aromatic spice	Root	Gingerol, turmeric, paradol, geraniol, geranial, borneol, linalool, camphene, zingerol, zingiberon	11.75 (dried ground root)	^25^ ^,^ ^27^
Chili pepper	Hot spice	Fruits	Capsaicin, hydrocapsaicin, ellagic acid, vanillic acid, p-coumaric acid, gallic acid, quercetin, luteolin, kaempferol, apigenin	9.30 (dried, ground fruit)	^29^ ^,^ ^30^
Turmeric	Aromatic spice	Root	Curcumins, essential oils, eugenol, carotene, ascorbic acid, caffeic, p-coumaric, protocatechuic, syringic, vanillic acid	8.40 (dried ground root)	^24^ ^,^ ^25^
Garlic	Vegetable (can be used as spice)	Bulb	Allicin, diallyl sulfide, diallyl disulfide, diallyl trisulfide, allyl isothiocyanate, *S*-allyl cysteine	5.90 (dried ground clove)	^25^
Cayenne pepper	Hot spice	Fruits	Capsorubin, capsanthin, capsolutein, capsaicin, hydrocapsaicin, zeaxanthin, cryptoxanthin, β-carotene	3.13 (dried ground fruit)	^31^
Other foods					
Dark chocolate	—	—	Phenolic acids (syringic, gallic, vanillic, coumaric, caffeic, etc), stilbenes, kaempferol, quercetin, procyanidins	5.8	^32^ ^,^ ^33^
Black currants	—	—	Gallic acid, sinapinic acid, caffeic acid, (epi)catechin, rutin, hesperidin	3.8	^34^ ^,^ ^35^
Onions	—	—	Quercetin, rutin, kaempferol, cyanidin, peonidin, pelargonidin	3.6	^36^ ^,^ ^37^
Broccoli	—	—	Quercetin, kaempferol, isorhamnetin, p-coumaric acid, sinapic acid, ferulic acid	1.4	^38^ ^,^ ^39^
Red grapes	—	—	(+)-Catechin, (−)-epicatechin, prodelphinidins, caffeic acid, caftaric acid, gallic acid	1.2	^40^ ^,^ ^41^

Abbreviation: GAE, Gallic acid equivalents.

Beyond their flavor-enhancing properties, spices are naturally rich concentrated sources of antioxidants including many phenolic compounds, with phenolic acids and flavonoids (flavones and flavonols) being the most abundant.^42–48^ Compared with other (poly)phenol-rich foods (eg, broccoli, dark chocolate, berries, grapes, onions, etc), spices generally contain far more (poly)phenolic compounds per unit weight (see Table 1).

(Poly)phenolic compounds in herbs and spices may be responsible for the beneficial effects discussed previously, in particular when it comes to oxidative stress. Oxidative stress, which can be defined as a high concentration of free radicals in cells and tissues, can be induced by several factors including gamma, ultraviolet (UV), X-ray radiation, psycho-emotional stress, polluted food, adverse environmental conditions, intensive physical exertion, smoking, alcoholism, and drug addiction.^49–53^ Chronic oxidative stress is known to contribute to the pathophysiology of many diseases, including cancer, metabolic disease, and acceleration of aging.^51^ In addition, some secondary products of lipid oxidation occurring as a part of oxidative stress processes (eg, malondialdehyde [MDA], 4-hydroxynonenal) can react with biological components such as protein, amino acids, and DNA.^53^ Malondialdehyde can be formed enzymatically and nonenzymatically as a result of these processes and may contribute to mutagenesis and carcinogenesis.^50^ Antioxidants from herbs/spices may be able help attenuate oxidative stress related damage and be used as ameliorative or preventive agents for several chronic diseases.^13^^,^^49–53^ For instance, phenolic acids and flavonoids, along with sulfur-containing compounds, tannins, alkaloids, phenolic diterpenes, and vitamins also present in spices, help to combat oxidative stress.^51^ Flavonoids in particular have the ability to scavenge free radicals by absorbing and neutralizing them, quenching singlet and triplet oxygen, decomposing peroxides, and forming complexes with catalytic ions, rendering them inactive.^52^^,^^53^ Compounds with antioxidant activity can also protect lipids and oils in foods against oxidative degradation. When added to foods, these compounds can help control rancidity development, slow the formation of toxic oxidation products, maintain nutritional quality, and extend the shelf-life of products.^53^

Herb and spice (poly)phenols, like all others, have low bioavailability, which may affect the extent of their health benefits. This also may be the reason for inconsistent results in some herb and spice interventions, including the inability to replicate in vitro experiment results in vivo.^55^ In addition, (poly)phenol content in spices/herbs is not consistent and may vary according to the use of the bioactive compound vs. the natural form, harvesting methods, storage time, processing, and cooking methods.^55^ Some spices are commonly consumed together as a mix and it is important to keep in mind that potential interactions between polyphenols from different spices in the mix may also affect each other’s bioavailability, consequently affecting the end health benefit.^55^

### Interactions With the Gut Microbiome

The concept of a dietary prebiotic was first introduced by Glenn Gibson and Marcel Roberfroid in 1995.^56^ The original definition described prebiotics as “a non-digestible food ingredient that beneficially affects the host by selectively stimulating the growth and/or activity of one or limited number of bacteria in the colon and thus improves host health.” This definition remained unchanged for over 15 years.^57^ According to this definition only several compounds qualified as a “prebiotic,” including short- and long-chain beta-fructans, lactulose, and galacto-oligosaccharides.^57^ In 2008, however, the International Scientific Association of Probiotics and Prebiotics (ISAPP) issued a revised definition, which stated that “dietary prebiotics are a selectively fermented ingredient that results in specific changes in the composition and/or activity of the gastrointestinal microbiota, thus conferring benefit(s) upon host health.”^58^ Additionally, to be classified as a prebiotic, the compound is required to be resistant to a low-pH stomach environment, be resistant to hydrolysis by mammalian enzymes, should not be absorbed in the gastrointestinal (GI) tract, can be fermented by the gut microbiota, and should be able to selectively stimulate growth and/or activity of gut bacteria.^58^ According to this definition, several noncarbohydrate compounds now qualify as prebiotics, including (poly)phenols.

Dietary (poly)phenols may contribute to the maintenance of gut homeostasis by stimulating the growth of generally beneficial bacteria such as bifidobacteria and lactobacilli, acting as prebiotics.^59^ They are also known to exhibit antioxidant and antimicrobial properties in the gut.^60^ (Poly)phenols are usually present in foods as glycosides and can be transformed into organic compounds by lipids, sugars, or organic acids.^61^ It is believed that as little as 5%-10% of dietary (poly)phenols are absorbed in the upper gut, while the remaining 90%-95% travel all the way to the colon (distal gut) where they are metabolized by the gut microbes. (Poly)phenol compound transformation by the gut microbes into various metabolites may affect the bioavailability of these compounds depending on the host gut resident microbes.^60^

(Poly)phenols from herbs/spices are known to have an impact on gut microbial composition and may undergo transformation from their unabsorbable parent form to an absorbable metabolite by hydrolyzation, ring cleavage, or reduction reactions.^62^ The specific metabolite formed, quantity of (poly)phenol consumed, and actions by the gut microbiota may play a role in determining the consequent health outcomes.^62^

## METHODS

A total of 8 human trials and 12 in vitro experiments were conducted at the University of California–Los Angeles (UCLA) Center for Human Nutrition between 2010 and 2024. These experiments included interventions with individual herbs/spices, such as cinnamon, chili pepper, and turmeric, as well as spice mixes. Results of these experiments were collated and summarized. Reference lists from the publications of these studies were manually scanned and relevant original, and review/meta-analysis, articles extracted. Several PubMed searches (between January 2025 and May 2025) were also conducted with the search words pertaining to each herb/spice and their effect on human health to expand on the discussed evidence.

## RESULTS AND DISCUSSION

### Health Benefits and Potential Underlying Mechanisms

A summary of herb/spice trials conducted at the UCLA Center for Human Nutrition described below is shown in Table 2.

**Table 2.** Overview of Spice/Herb Studies Conducted at the UCLA Center for Human Nutrition

Study (year)	Objective	Participants	Study design	Duration	Intervention/control	Results
Cinnamon						
Wang et al. (2020)^63^	To investigate the acute effect of cinnamon addition to a typical American breakfast on postprandial glucose, insulin, C-peptide, and glucagon	*n* = 32 adults with normal weight, overweight/obesity	RCT, crossover, open-label, pilot	2-wk run-in + 2 test meals 1 wk apart	1/2 cup dry instant oatmeal + 1 cup 2% milk served with or without 6 g of ground cinnamon	Normal-weight cinnamon group: - ↓ Postprandial glucagon niAUC_0-180_ and glucagon levels at 60, 90, and 120 min - ↓ C-peptide at 30 min Overweight/obese cinnamon group: - ↓ Postprandial insulin niAUC_0-180_ and serum insulin at 30 min - ↑ Blood glucose at 60 min All participants together cinnamon group: - ↓ Postprandial insulin and glucagon niAUC_0 − 180min_ - ↓ Serum insulin and C-peptide at 30 min - ↑ Blood glucose at 60 min
Zelicha et al. (2024)^64^	To determine the effect of daily cinnamon spice supplementation in an amount commonly used for seasoning on glucose concentrations	*n* = 18 adults with prediabetes and obesity	RCT, crossover	4 wk	4 g/d of cinnamon or placebo	Cinnamon group: - ↓ In 24-h glucose, netAUC for glucose, glucose peaks - ↑ GDIP during OGTT - ↓ TGs - ↓ RA of *Terrisporobacter* and *Dialister* - ↑ RA of *Methanobrevibacter* - No difference in GI symptoms between groups
Chili pepper						
Lee et al. (2010)^65^	To examine DCT effects on both adaptive thermogenesis and to determine whether DCT would increase PPEE in response to a 4000kcal/60 g protein liquid test meal	*n* = 33 healthy men and postmenopausal women	RCT, parallel	4 wk	3 mg placebo or 3 mg/9 mg DCT + VLCD plan: 8 servings/d of very-low-kcal protein shake providing 100 kcal and 15 g of high-quality protein + 40 mEq of KCl	- No difference between groups in weight loss, fat mass, or BMR - ↑ PPEE at day 0 in response to test meal in all patients - ↑ PPEE in DCT group vs control at day 28 - ↑ RQ in placebo group only at day 28
Turmeric						
Wang et al. (2014)^66^	To investigate whether a combination of 3 natural products (arctingenin, -[-]EGCG, and curcumin) increases the chemopreventive potency of individual compounds	NA	In vitro (LNCaP and MCF-7 cell lines)	48 h	5-10 µM curcumin, 1 µM arctingenin, and 40 µM EGCG alone or in combination	- In both cell lines treatment with mixture synergistically: ↑ antiproliferative effect - The strongest effects on cell cycle arrest and apoptosis were achieved by combining all 3 compounds in both cell lines - The combination treatment synergistically ↑ ratio of Bax to Bcl-2 proteins, ↓ activation of NF-κB, PI3K/Akt, and STAT3 pathways and cell migration
Small et al. (2018)^67^	To study the effect of curcumin on memory and to explore its impact on brain amyloid and tau accumulation using FDDNP-PET	*n* = 40 nondemented adults	RCT, parallel	18 mo	Bioavailable form of curcumin (Theracurmin) containing 90 mg of curcumin/2× daily	- Significant improvement baseline vs endpoint in curcumin group for verbal/visual memory, secondary attention, Beck Depression Inventory score FDDNP-PET: - No between-group differences in amygdala but FDDNP binding levels ↓ in curcumin group - Changes in hypothalamic binding were significantly different between groups
Spice mixes						
Li et al. (2010)^68^	To determine the effects of adding a (poly)phenol-rich spice mixture containing rosmarinic acid to hamburger meat before cooking to establish the effects on the absorption of cytotoxic lipids as determined by measuring MDA concentrations in plasma and urine	*n* = 10 healthy adults	RCT	2 test meals on 2 different days	- A cooked ground beef burger (control) - A ground beef burger seasoned with a spice mixture (clove, cinnamon, oregano, rosemary, ginger, black pepper, paprika, garlic powder)	- 71% ↓ MDA content in cooked burger + spice - ↑ Plasma/urine MDA after control burger - ↓ Urine MDA after burger + spice - Plasma MDA trended down after burger + spice
Henning et al. (2011)^69^	To compare antioxidant capacity of 10 herbs and spices in dry, fresh, and blended herb paste form via TEAC assay	NA	Chemical analyses	NA	10 herbs (basil, oregano, Italian seasoning, garlic, ginger, chili pepper, parsley, cilantro, dill)	- Except for garlic in dry form and lemon grass in fresh and paste form, all herbs retained considerable antioxidant activity - Oregano had the strongest AC in fresh and dry form - ↓ AC in dry form vs. fresh in garlic, chili, dill, oregano, parsley - Phenolic content correlated well with AC in dry and fresh form but not in paste form - ↓ Content of representative marker compounds in dry herbs vs fresh for basil, chili, dill, garlic, ginger; equal in parsley and ↑ in oregano and cilantro - ↓ Marker compound concentration in paste vs fresh in all herbs - No overall correlation between concentration of chemical markers and AC
Li et al. (2013)^70^	To investigate whether spice (poly)phenols reduce GI and systemic formation of cytotoxic lipid peroxidation products and increase NO formation, thereby leading to improved endothelial vascular function	*n* = 11 men with type 2 diabetes	RCT	2 test meals, 1 wk apart	- Ground beef seasoned with salt only - Ground beef seasoned with salt and spice mixture	- No difference in postprandial glucose, insulin, TGs - 31% ↓ in urine MDA and creatinine in spice group - ↓ in PAT score 2 h after burger consumption in control and ↑ in spice group - No differences in nitrate/nitrite
Zhang et al. (2015)^71^	To determine the effect of combining curcuminoids and piperine on the antioxidant activity and lipid peroxidation during the preparation of hamburger meat	NA	Chemical analyses	NA	48 fried hamburger patties with black pepper and turmeric; spices used in comparison: oregano, garlic, oregano/garlic, turmeric, paprika, spice mixture	- Recovery of curcuminoids after cooking was 60% independent of the amount of turmeric added - Addition of increasing amounts of black pepper did not affect the recovery of curcuminoids - Recovery of piperine increased as more black pepper was added, regardless of the amount of curcuminoids present - Increasing amounts of curcumin led to ↓ lipid peroxidation during cooking - When black pepper was added to turmeric it resulted in a synergistic ↓ of MDA formation vs. same amount of curcumin without black pepper - Antioxidant activity of black pepper was very small vs. turmeric - Piperine did not exhibit any DPPH scavenging activity, while curcumin was shown as the main antioxidant contributor in turmeric powder
Li et al. (2015)^72^	To determine the feasibility of evaluating the impact of spices added to vegetables on amount and rate of vegetable consumption under controlled conditions	*n* = 20 generally healthy overweight adults	RCT, pilot	6 study visits	Broccoli, cauliflower, spinach with or without spice (each had its own custom blend of spices formulated specifically to maximize taste)	- ↑ Consumption of broccoli and duration of eating ad libitum when spice was added (rate of eating did not change) - No differences in the amounts, duration, rate of cauliflower or spinach consumed with or without spices - VAS measures of mood, appetite, and taste were not significantly different between plain vs. spice test meals
Lu et al. (2017)^73^	To investigate major chemical constituents, antioxidant activity, and in vitro effect of 7 spice extracts on the growth of 33 beneficial *Bifidobacterium* spp and *Lactobacillus* spp to establish their antimicrobial activity against 88 intestinal, pathogenic, and toxigenic bacterial strains	NA	Chemical analyses	NA	7 spices were tested: black pepper, cayenne pepper, cinnamon, ginger, Mediterranean oregano, rosemary, turmeric	- Most spice extracts contained either moderate or high content of spice-specific phytochemicals - Various spice extracts (except for turmeric) stimulated growth of either *Lactobacilli* or *Bifidobacteria* - All spices exhibited high inhibitory activity against *Ruminococcus* species but minimal or no activity against selected strains of *Bacteroides*, *Finegoldia*, *Escherichia coli*, *Salmonella*, and *Staphylococcus* - Oregano, black pepper, cayenne pepper, and ginger exhibited prebiotic-like effects by simultaneously promoting the growth of beneficial bacteria and suppressing pathogenic bacteria
Lu et al. (2019)^74^	To investigate the effects of mixed spices at culinary doses on the gut microbiota and production of SCFAs	*n* = 31 healthy adults	RCT, parallel	2 wk	Intervention: 5 g spice mix (cinnamon, oregano, ginger, black pepper, cayenne pepper) capsule vs maltodextrin capsule	- α-Diversity and β-diversity were not affected by spices - 6% ↑ in *Firmicutes* in control and 10% ↓ in spice group - 19% ↑ in *Bacteroidetes* in spice group and 14% ↓ in controls - 26 OTUs were significantly different between groups - No differences in SCFAs between groups

Abbreviations: AC, antioxidant capacity; BMR, basal metabolic rate; DCT, dihydrocapsiate; DPPH, 2,2-diphenyl-1-picrylhydrazyl; EGCG, epigallocatechin gallate; FDDNP-PET, 2-(1-{6-[(2-[F-18]fluoroethyl)(methyl)amino]-2-naphthyl}ethylidene)malononitrile positron emission tomography; GDIP, glucose-dependent insulinotropic-polypeptide; GI, gastrointestinal; MDA, malondialdehyde; NA, not available; NF-κB, nuclear factor–kappa B; netAUC, net area under the curve; niAUC, net incremental area under the curve; NO, nitric oxide; OGT, oral-glucose-tolerance test; OTU, operational taxonomic unit; PI3K, phosphatidylinositol 3-kinase; PPEE, postprandial energy expenditure; RA, rheumatoid arthritis; RCT, randomized controlled trial; RQ, respiratory quotient; SCFA, short-chain fatty acid; STAT3, signal transducer and activator of transcription 3; TEAC, Trolox Equivalent Antioxidant Capacity; TG, triglyceride; UCLA, University of California–Los Angeles; VAS, visual analog scale; VLCD, very-low-calorie diet.

#### Cinnamon

##### Evidence from the UCLA Center for Human Nutrition

A study involving cinnamon spice was conducted at the UCLA Center for Human Nutrition in 2021.^63^ In this trial, 32 individuals consumed 2 test meals 1week apart. The test meals were either ½ cup of dry instant oatmeal + 1 cup of 2% milk served with or without 6 g of ground cinnamon. This was a randomized, controlled, crossover, open-label pilot trial. The purpose of this trial was to investigate the acute effect of cinnamon addition to a typical American breakfast on postprandial glucose, insulin, C-peptide, and glucagon in normal-weight and overweight/obese participants. Interestingly, the obtained results differed between normal-weight and overweight/obese individuals. In normal-weight individuals the postprandial glucagon niAUC_0-180 min_ and glucagon levels at 60, 90, and 120 minutes postprandially were lower after the test meal with cinnamon vs. the test meal without cinnamon. In addition, C-peptide at 30 minutes was decreased with cinnamon, but there was no difference in niAUC_0-180 min_ for glucose, insulin, and C-peptide between groups. In individuals with overweight/obesity, postprandial insulin niAUC_0-180 min_ and serum insulin at 30 minutes were lower with cinnamon, while blood glucose at 60 minutes was higher in the cinnamon group vs the control group. When data from all participants were combined for analysis, the results showed that postprandial insulin and glucagon niAUC_0 − 180 min_ remained lower after the meal with cinnamon compared with the test meal without cinnamon. Insulin and C-peptide levels at 30 minutes were lower, while glucose levels at 60 minutes were higher after the meal with cinnamon compared with the test meal without cinnamon.

A more recent study by Zelicha et al.^64^ conducted at the UCLA Center for Human Nutrition in 2024 explored the effect of daily cinnamon spice supplementation in an amount commonly used for seasoning (4 g/d) on glucose concentrations as well as the gut microbiome in adults with obesity and prediabetes. This was a randomized, controlled, double-blind, crossover trial enrolling 18 participants. Participants were randomly assigned to consume cinnamon or placebo for 4 weeks, followed by a 2-week washout period, and then crossed over to the other intervention for an additional 4 weeks. Glucose changes were measured with continuous glucose monitoring, as well as an oral-glucose-tolerance test (OGTT) immediately following ingestion of cinnamon or placebo. Digestive symptom logs were also collected. The major results in this study were that 24-hour glucose was lower in the cinnamon group compared with the control group at the study’s endpoint. In addition, netAUC for glucose was also lower in the cinnamon group. Glucose-dependent insulinotropic-polypeptide (GDIP) concentrations increased during OGTT after cinnamon but not after the placebo. In terms of the gut microbiome, α-diversity was different between the groups at endpoint. There were also changes in the relative abundance of several microbial genera, including *Terrisporobacter* and *Dialister* (reduced with cinnamon), as well as *Methanobrevibacter* (increased with cinnamon).

##### Evidence from other research groups and potential mechanisms of action

This evidence is consistent with what has been reported by other research groups. For instance, several studies showed dose-dependent glucose-lowering effects of cinnamon in healthy individuals as well as in individuals with type 2 diabetes.^75–77^ Other potential health benefits of cinnamon consumption include protection against major respiratory and GI pathogens, anti-inflammatory and antioxidant properties, as well as hepato- and neuroprotective benefits.^78–82^

Potential underlying mechanisms of cinnamon health benefits include regulation of anti- and proinflammatory gene expression and inhibition of cyclooxygenase-2 (COX2) and inducible nitric oxide synthase (iNOS).^83^ Cinnamaldehyde, a major bioactive compound in cinnamon, can also act on multiple signaling pathways such as peroxisome proliferator–activated receptors (PPARs), AMP-activated protein kinase (AMPK), phosphatidylinositol 3-kinase/insulin receptor substrate 1 (PI3K/IRS-1), retinol-binding protein 4 (RBP4), glucose transporter type 4 (GLUT4), extracellular signal–regulated kinases/c-Jun N-terminal kinases/p38 mitogen-activated protein kinases (ERK/JNK/p38MAPK), transient receptor potential ankryrin 1 (TRPA), ghrelin, and nuclear factor erythroid 2–related factor 2 (Nrf2) pathways.^83^

#### Red Pepper

Capsaicin from chili pepper is known to stimulate thermogenesis through a central nervous mechanism, but at doses required to observe this metabolic effect, serious GI side effects occur.^84^ Previous evidence suggests that capsinoids (eg, dihydrocapsiate [DCT]) found in nonpungent CH-19 sweet red pepper share the positive metabolic characteristics of capsaicin without inducing GI side effects.^84^^,^^85^ Dihydrocapsiate, like capsaicin, stimulates transient receptor potential vanilloid 1 (TRPV1) receptors in the gut, which activate the sympathetic nervous system, potentially increasing lipogenesis and thermogenesis.^85^ Extracts from CH-19 sweet pepper may have the potential to increase thermogenesis, oxygen consumption, sympathetic nervous system activation, and weight loss.^86^^,^^87^

##### Evidence from the UCLA Center for Human Nutrition

The purpose of a study conducted at the UCLA Center for Human Nutrition in 2010 aimed to examine DCT effects on adaptive thermogenesis and to determine whether DCT increases postprandial energy expenditure (PPEE) in response to a 400-kcal/60 g protein liquid test meal.^65^ Adaptive thermogenesis in this study was induced by caloric restriction with a high-protein very-low-calorie diet (VLCD). The VLCD consisted of 8 servings of a very-low-calorie (100 kcal) high-protein (15 g) shake daily. This was a randomized double-blinded human trial involving 33 healthy men and postmenopausal women with overweight/obesity. Participants were asked to consume either 3 mg placebo or 3 mg or 9 mg DCT for 4 weeks. Vital signs, anthropometric measurements (including body composition), indirect calorimetric measurements, as well as blood samples were collected. As a result of this intervention, participants in all 3 groups lost a significant amount of weight, but there was no difference between groups. There was an increase in PPEE at 1 hour after test meal consumption at endpoint in both the placebo and DCT groups, but the difference was significant only in the 9-mg DCT group. At endpoint there was also an increase in postprandial respiratory quotient in the placebo group only and this increase was significantly different compared with both DCT groups (3 and 9 mg). The study concluded that there may be an increase in thermogenesis and fat oxidation secondary to administration of DCT.

##### Evidence from other research groups and potential mechanisms of action

A recent triple-blinded, placebo-controlled, randomized crossover study enrolling 24 sedentary men (age = 40.2 ± 9.2 years; BMI = 31.6 ± 4.5 kg/m^2^) investigated whether the ingestion of 12 mg of DCT would increase resting energy expenditure (EE) and specifically fat oxidation (FATox) during an acute bout of aerobic exercise.^88^ Indirect calorimetry was used to evaluate respiratory gas exchange, and several serum marker concentrations (eg, glucose, triglycerides, nonesterified fatty acids, etc) were also assessed. Surprisingly, the results demonstrated that there were no significant differences between DCT and placebo conditions in the EE and FATox groups during exercise.^88^ No differences were observed in serum biomarkers either. Another study attempted to determine the effects of DCT on resting metabolic rate (RMR).^89^ This was a double-blind parallel trial involving 78 healthy adults. The dosage of DCT and duration were equal to the doses and duration in the Lee et al. study^65^ described above: 0, 3 and 9 mg for 28 days. The RMR in this study was measured via indirect calorimetry for 30 minutes before and 120 minutes after ingestion of DCT. Results demonstrated that there was no difference between groups after acute DCT ingestion.^89^ There was a borderline effect at endpoint in the group receiving 3 mg vs placebo and no effect in the 9-mg DCT group. However, when data from both intervention groups were combined, the thermic effect of DCT reached statistical significance (*P* < .05). Notably, fat oxidation was unaffected by DCT.^89^

#### Turmeric

##### Evidence from the UCLA Center for Human Nutrition

One of the studies conducted at the center by Wang et al^66^ in 2014 aimed at investigating whether a combination of 3 natural products—arctigenin (Arc), a novel anti-inflammatory lignan from the seeds of *Arctium lappa*, green tea polyphenol (-)-epigallocatechin gallate (EGCG), and curcumin (Cur)—increases the chemo-preventive potency of individual compounds. LNCaP prostate cancer and MCF-7 breast cancer cells were treated with 5–10 µM Cur, 1 µM, Arc and 40 µM EGCG alone or in combination for 48 hours. Results indicated that in both cell lines treatment with the mixture of all 3 compounds synergistically increased the antiproliferative effect. Similarly, the strongest effects on cell cycle arrest and apoptosis were achieved by combining all 3 compounds in both cell lines. Moreover, the combination treatment significantly increased the ratio of Bax to Bcl-2 proteins, decreased activation of nuclear factor–kappa B (NF-κB), PI3K/Akt, and signal transducer and activator of transcription 3 (STAT3) pathways and cell migration compared with individual treatments. These results warrant in vivo studies to confirm the efficacy of this novel regimen by combining Arc, EGCG, and Cur to enhance chemoprevention in both cell types.^66^

Another study utilizing curcumin was conducted at the center in 2018 by Small et al.^67^ The purpose was to study the effect of curcumin on memory in nondemented adults and to explore its impact on brain amyloid and tau accumulation using 2-(1-{6-[(2-[F-18]fluoroethyl)(methyl)amino]-2-naphthyl}ethylidene)malononitrile positron emission tomography (FDDNP-PET). This was an 18-month, double-blind, placebo-controlled, parallel trial enrolling 40 middle-aged and older adults. A bioavailable form of curcumin (Theracumin) containing 90 mg of curcumin consumed twice daily was used as the intervention. Primary outcomes of interest included verbal and visual memory evaluations, including the Buschke Selective Reminding Test and Brief Visual Memory Test among others. Secondary outcomes focused on attention tests and FDDNP-PET signals in various brain regions (eg, amygdala, hypothalamus, etc.). Participants in the curcumin group showed significant improvement from baseline in both verbal and visual memory, as well as attention and depression (Beck Depression Inventory) scores. Furthermore, amygdala FDDNP binding levels declined significantly from baseline in the curcumin group, while there were no changes in the control group. There were no differences between groups at endpoint, however. In contrast to this result, hypothalamus FDDNP binding was significantly different between the curcumin and control groups after 18 months of intervention. Overall, these findings suggest that daily oral ingestion of a bioavailable form of curcumin may help improve memory performance in humans.^67^ Moreover, such daily oral curcumin consumption may lead to less neuropathological accumulation in several brain regions, including the amygdala and hypothalamus.^67^

##### Evidence from other research groups and potential mechanisms of action

A recent double-blind randomized controlled trial (RCT) demonstrated that oral administration of curcumin and piperine together for symptomatic COVID-19 therapy might aid in significantly reducing mortality and morbidity.^90^ In addition to conventional COVID-19 treatment, patients in the control group received a probiotic and patients in the intervention group received 525 mg of curcumin and 2.5 mg of piperine twice daily. Patients with mild, moderate, and severe symptoms who received curcumin/piperine tablets showed early symptomatic recovery and better clinical outcomes compared with the control group.^90^ Shorter hospitalization durations and fewer deaths were also observed in the curcumin/piperine group compared with controls.^90^ Another study tested 180 mg of Theracumin (commercial compound containing curcumin) daily for 8 weeks in 50 patients with knee osteoarthritis.^90^ Interestingly, knee pain significantly decreased in the Theracumin group compared with the controls at endpoint.^90^ Other studies demonstrated that curcumin may also have cardioprotective properties. For instance, in a human trial conducted by Yang and colleagues^91^ in 2014, 650 mg of curcumin extract were given to participants with metabolic syndrome 3 times daily for 12 weeks (intervention group, *n* = 33; placebo group: *n* = 32). At endpoint after the curcumin extract consumption, levels of high-density-lipoprotein (HDL) cholesterol significantly increased and the levels of low-density-lipoprotein (LDL) cholesterol significantly decreased from baseline. Weight and glucose levels remained unchanged, however. Overall, these results indicate that daily curcumin consumption may be an effective alternative for modifying cholesterol-related parameters in patients with metabolic syndrome.^91^

In terms of mechanism of action, curcumin demonstrated potent antioxidant activities comparable to activities of vitamins C and E. Curcumin has the potential to scavenge reactive oxygen and nitrogen species, including superoxide anion, hydroxyl radicals, and nitrogen dioxide.^92^ It also exhibited capability for inhibiting lipid peroxidation and activating genes coding for antioxidant enzymes, including superoxide dismutase, catalase, glutathione peroxidase, and *S*-glutathione transferase.^92^ In addition, curcumin can inhibit the activity of NF-κB transcription factor, arrest cell cycle at its different stages, and induce apoptosis in cancer cells.^82^

#### Spice Mixes: Black Pepper, Cayenne Pepper, Turmeric, Oregano, Garlic, Paprika, Rosemary, Ginger

##### Evidence from the UCLA Center for Human Nutrition

Several studies at the UCLA Center for Human Nutrition over the years have explored the effects of spice mixtures on human health. One of them was aimed at determining the effects of adding a (poly)phenol-rich spice mixture to hamburger meat before cooking to establish the effects on the absorption of cytotoxic lipids as determined by measuring MDA concentrations in participants’ plasma and urine.^68^ This was a prospective, randomized pilot study involving 10 healthy volunteers. Volunteers in this trial in random order consumed 2 different test meals consisting of a cooked ground beef burger (control) and a ground beef burger seasoned with a spice mix during cooking (treatment). Results indicated that MDA content in the cooked burger was significantly decreased by 71% with vs without the addition of the spice mix. In addition, MDA in participants’ plasma/urine increased after the consumption of the control burger, while it trended down in plasma and was significantly lower in urine after consumption of the spiced burger. In this study, the stomach was acting as a bio-generator and spices reduced the effects of meat consumption on MDA production through antioxidant effects.^68^

In 2011, Henning et al.^69^ conducted another study that aimed at comparing antioxidant capacity of 10 herbs and spices (basil, oregano, Italian seasoning, garlic, ginger, chili pepper, parsley, cilantro, dill, lemon grass) in dry, fresh, and blended herb paste form using the Trolox Equivalent Antioxidant Capacity (TEAC) assay. In addition, total phenolic content and chemical markers in all 3 formulations were evaluated. Interestingly, except for garlic in dry form and lemon grass in fresh and paste form, all herbs/spices retained considerable antioxidant activity across the 3 different formulations. Oregano had the strongest antioxidant capacity in fresh and dry form, while antioxidant capacity significantly decreased in dry vs. fresh form in garlic, chili pepper, dill, oregano, and parsley. In addition, phenolic content correlated well with antioxidant capacity in dry and fresh, but not in paste, form of herbs/spices tested in this experiment. The comparative analysis of the content of representative marker compounds (eg, rosmarinic acid in basil and oregano, allicin in garlic, etc.) between the 3 formulations revealed that it was generally lower in dry and paste vs. fresh formulations. Notably, allicin was detected only in the freshly ground garlic but not in the dry or paste forms.^69^

Another study conducted at the center in 2013 aimed at investigating whether spice (poly)phenols reduced the intestinal and systemic formation of cytotoxic lipid peroxidation products and increased nitric oxide (NO) formation, thereby leading to improved endothelial vascular function in men with type 2 diabetes.^70^ This was a randomized crossover trial enrolling 11 individuals. Two test meals 1 week apart were consumed: one of them consisted of ground beef seasoned with salt only and the other was ground beef seasoned with salt and a spice mixture. Interestingly, the results demonstrated that there was no difference in postprandial glucose, insulin, triglycerides, or nitrate/nitrite after consumption of both meals. Changes over time were also not significant. However, urine MDA and creatinine were reduced by 31% in the group that consumed the test meal with the added spice mixture. In addition, 2 hours after the burger consumption, peripheral arterial tonometry (PAT) score, a measure of endothelial function, decreased after the consumption of the control burger, while it increased after consumption of the burger with added spices.^70^

To continue investigating the mechanism underlying the effects of the spice mixture during cooking, a chemical analysis of ground beef patties was conducted.^71^ Hamburger patties were spiced prior to cooking with either black pepper and turmeric or other spices, including garlic, oregano, garlic + oregano, turmeric, and paprika. Free radical scavenging capacity, quantification of curcuminoids in turmeric powder, and piperine in black pepper powder, as well as MDA concentration in the burgers after cooking were carried out. The results showed that recovery of curcuminoids after cooking was 60%, independent of the amount of turmeric added, while the addition of increasing amounts of black pepper did not affect the recovery of curcuminoids from the burgers after cooking. Piperine recovery correlated with the amount of black pepper added, regardless of the amounts of curcuminoids present. Interestingly, increasing amounts of curcumin led to reduced amounts of lipid peroxidation during cooking. This did not occur for black pepper, however. In addition, when black pepper was added to turmeric powder at 3 and 6 g per patty, it resulted in a synergistic inhibition of MDA formation compared with the same amount of curcumin used without black pepper. Piperine did not exhibit any DPPH scavenging activity, while curcumin was shown as the main antioxidant contributor in turmeric powder; radical scavenging capacity was not changed when black pepper and turmeric were added to the patty. Piperine is included in many studies of turmeric as a way of increasing bioavailability, adding significance to our studies demonstrating a different mechanism of effects of piperine and curcumin during cooking.^71^

The impact of adding spices to vegetables as a strategy for increasing the palatability and consumption of vegetables was investigated at the center.^72^ Spices were added to vegetables, including broccoli, cauliflower, and spinach, and the amount and rate of vegetable consumption under controlled conditions measured using a Universal Eating Monitor (UEM). Pre- and postconsumption taste and appetite ratings were performed. This was a prospective randomized pilot study including 20 individuals. Participants in this study consumed broccoli, cauliflower, and spinach, with or without spices (each of these vegetables had its own custom blend of spices formulated specifically to maximize taste). Six different vegetable combinations were consumed at 6 different occasions. Measurements were taken via UEM, and mood was assessed using a computer-based visual analog scale (VAS). The results demonstrated that the consumption of broccoli increased when the spice mixture was added. Interestingly, the duration of eating broccoli ad libitum was also increased with the addition of spices. On the other hand, the rate of eating broccoli remained unchanged. For other vegetables, including spinach and cauliflower, there were no differences observed in the amount, duration, or rate of consumption with or without spices. In addition, VAS measures of mood, appetite, and taste were not significantly different between plain vs spice test meals for all 3 vegetables.^72^

Later, 2 other studies at the center investigated the effects of spice mixtures on the gut microbiota composition and functionality.^73^^,^^74^ To test the hypothesis of spices being able to modulate the gut microbiota, researchers at the center performed a comprehensive chemical analysis to investigate major chemical constituents, antioxidant activity, and in vitro effect of a combination of 7 spice extracts on the growth of 33 beneficial *Bifidobacterium* spp and *Lactobacillus* spp.^73^ The antimicrobial activity of these beneficial strains was tested against 88 intestinal, pathogenic, and toxigenic bacterial strains (isolated from patients at the Greater Los Angeles Veteran Affairs Hospital), harkening back to the traditional uses of spices as food preservatives and medicines. The spices tested were black pepper, cayenne pepper, cinnamon, ginger, Mediterranean oregano, rosemary, and turmeric. The results indicated that most of these spice extracts contained either moderate or high contents of spice-specific phytochemicals (the majority were polyphenols and phenolic acids). In addition, various spice extracts (except for turmeric) stimulated growth of either lactobacilli or bifidobacteria. Interestingly, all 7 spices exhibited high inhibitory activity against *Ruminococcus* species but minimal or no activity against selected strains of *Bacteroides*, *Finegoldia*, *Escherichia coli*, *Salmonella*, and *Staphylococcus*. *Fusobacterium* spp were susceptible to the cinnamon extract and *Fusobacterium necrophorum* appeared to be susceptible to the oregano as well as the rosemary extracts. Moreover, cinnamon extract exhibited a modest activity against *Clostridioides difficile*, while cinnamon, rosemary, and turmeric extracts were effective against a few other *Clostridium* strains. It is important to point out that, among the spices investigated, oregano, black pepper, cayenne pepper, and ginger possessed prebiotic-like effects by promoting the growth of beneficial bacteria on one hand and suppressing pathogenic bacteria on the other, suggesting their potential role in the regulation of intestinal microbiota and the enhancement of GI health.^73^

The results from the abovementioned study inspired researchers at the center to conduct another study—this time a clinical trial aimed at investigating the effects of mixed spices (cinnamon, oregano, ginger, black pepper, and cayenne pepper) at culinary doses (5 g) consumed over 2 weeks in a standardized 5 g capsule on the composition of gut microbiota and production of short-chain fatty acids (SCFAs) in healthy participants compared with a placebo maltodextrin capsule.^74^ This trial was a randomized, placebo-controlled, double-blind pilot study enrolling 31 participants. The results demonstrated that α-diversity and β-diversity were not affected by the spice mixture (richness and evenness). At the same time, there were some differences in microbial relative abundances. For instance, the relative abundance of *Firmicutes* increased by 6% in the control group and decreased by 10% in the spice group, while the relative abundance of *Bacteroidetes* increased by 19% in the spice group and decreased by 14% in controls. In addition, 26 operational taxonomic units (OTUs) were significantly different between groups (21 increased and 5 decreased). Specifically, there was a significant enrichment in *Bifidobacterium animalis*, *Bacteroidetes fragilis*, and *Lactobacillus* genera and a significant reduction in the *Clostridium* genus. On the one hand, there were no differences in fecal SCFA levels. On the other hand, there was a negative correlation between propionate and *Firmicutes* and a trend of a positive correlation between propionate and *Bacteroidetes* in the spice group compared with the control group.^74^

##### Evidence from other research groups and potential mechanisms of action

###### Black pepper

Evidence from other research groups demonstrated that, in addition to gut microbiota–modulating properties, piperine may also be able to exhibit antiproliferative effects in MCF-7 cell lines.^93^ In addition, it may be effective in improving cardiometabolic biomarker levels. For instance, melanocortin-4 (MC-4) is a hypothalamic neuropeptide that, when bound to its receptor, has the ability to control the feeding mechanism, thereby potentially regulating obesity.^94^ In addition, other studies indicated that piperine may be able to cross the blood–brain barrier, potentially exhibiting neuroprotective effects for Alzheimer’s disease, Parkinson’s disease, and cognitive impairment.^95^^,^^96^

The potential mechanisms of action may be implied from several in vitro experiments. For instance, in a recent arthritis model, piperine treatment reduced COX-2, NOS-2, and NF-κB proinflammatory cytokines (eg, interleukin-6 [IL-6], matrix metalloproteinase-13, activator protein-1, and prostaglandin E2) and significantly reduced arthritic and nociceptive symptoms.^97^ Similar results were observed in a cerebral ischemia-reperfusion–induced inflammation rat model, where piperine treatment affected the same inflammatory markers as well as tumor necrosis factor-α (TNF-α).^98^ Additional evidence also shows that piperine may be able to protect against oxidative damage by quenching free radicals and ROS, as well as lower lipid peroxidation.^99^^,^^100^

###### Cayenne pepper

In addition to the health benefits of capsaicin observed in the studies at the UCLA Center for Human Nutrition, significant pain relief was reported in a recent clinical trial involving 70 patients with osteoarthritis and 31 patients with rheumatoid arthritis (RA) receiving capsaicin for 4 weeks.^101^

Moreover, capsaicin may also be able to inhibit formation of PGE2 and leukotrienes from arachidonic acid in macrophages and modulate release of proinflammatory factors, resulting in the stimulation of the TRPV1 channel.^102^ Additional capsaicin mechanisms of action include suppressing production of PGE2 via inhibition of COX-2 enzyme expression, suppressing iNOS, and inhibiting the signaling pathway linking proinflammatory stimuli with cyclooxygenase activation, including the suppression of nuclear translocation of NF-κB.^103–106^

###### Turmeric

Turmeric is rich in curcuminoids, with the most prominent example being curcumin. Several prior experiments showed that curcumin can ameliorate oxidative stress—the primary cause of inflammation.^107^^,^^108^ Strikingly, efficacy was found to be comparable to the standard anti-inflammatory drug phenylbutazone.^109^

Curcumin has shown the potential to protect cardiac function, vascular health, and lipid profiles in animals and humans.^110^ It may also be helpful for patients with inflammatory bowel disease and irritable bowel syndrome.^82^ In addition, it has been demonstrated to be effective against the development of hepatic steatosis and its progression to steatohepatitis.^111^ It may help maintain healthy joint function.^112^ Moreover, curcumin has been used as an analgesic and anti-inflammatory agent for the management of arthritis.^82^ It may help with blood glucose control, weight management, chemoprevention, and depressive moods.^82^^,^^111^^,^^112^

In terms of possible mechanisms of action, in vitro evidence suggests that curcumin can scavenge free radicals, inhibit lipid peroxidation and LDL oxidation, and protect DNA from oxidative damage. It may also be able to inhibit COX-2, prostaglandins, leukotrienes, TNF-α, and NF-κB.^91^

###### Oregano

Oregano extract may be able to exhibit antidiabetic properties by inhibiting α-glucosidase and aldose reductase and preventing albumin glycation in vitro.^113^ In addition, extracts from cultured shoot tissue of various clonal lines of oregano inhibited pancreatic amylase activity by between 9% and 57%.^113^ Oregano extract may also be able to antagonize peroxisome PPAR-γ and increase insulin-stimulated glucose uptake by adipocytes without stimulating adipocyte differentiation.^114^ Moreover, oregano extract increased endothelial NOS (eNOS) activity in umbilical vein endothelial cells and inhibited iNOS protein expression and NO production in lipopolysaccharide (LPS)-activated macrophages in a dose-dependent manner.^114^ In another in vitro study when LPS-stimulated macrophages were exposed to oregano extract they produced less IL-6 and TNF-α, which may mean that it has the potential lower inflammation.^115^

###### Garlic

Organosulfur compounds in garlic have exhibited lipid-lowering, antioxidant, anti-inflammatory, anticancer, glucose-lowering, and hepatoprotective properties.^116^ A recent literature review reported its potential effects on oxidative stress, cardiovascular disease, inflammation, and cancer.^116^ A recent human trial demonstrated that 200 mg of raw crushed garlic consumption for 4 weeks lowered several metabolic risk factors, including blood pressure, blood glucose, and triglycerides as well as LDL cholesterol.^117^

Organosulfur compounds in garlic may also be effective in suppressing benzo(a)pyrene tumorigenesis correlated with their ability to suppress nicotinamide adenine dinucleotide phosphate (NADPH):quinone oxidoreductase.^118^ In addition, a recent in vitro experiment demonstrated that garlic extract and allicin efficiently scavenged exogenously generated hydroxyl radicals in a dose-dependent fashion.^119^^,^^120^

###### Paprika

Carotenoids in paprika, like carotenoids from other foods, can accumulate in the liver or adipose tissue, and later be incorporated into plasma lipoproteins and serve as strong scavengers of free radicals in the body. Their other activities include reduction in cholesterol levels, as well as exerting antioxidant and anti-inflammatory effects. In addition, a recent in vitro experiment demonstrated that red paprika extract and its main carotenoid, capsanthin, prevented phosphorylation of Cx43 protein induced by hydrogen dioxide (H_2_O_2_) treatment in rat liver epithelial cells.^121^ They also appeared to be protective against H_2_O_2_-induced damage to gap-junction intercellular communication in these cells. Suppression of reactive oxygen species (ROS) formation was also reported.^121^

###### Rosemary

In vitro evidence shows that rosemary extract may have antioxidant effects by suppressing NF-κB, IL-1β, and COX-2.^122^ It may also be able to activate PPAR-γ, thereby regulating cellular functions and metabolism and leading to lower blood glucose levels.^123^ A recent double-blind RCT assessed the effects of rosemary powder added to tomato juice on cognitive function in 27 elderly individuals.^123^ The rosemary doses used ranged between 750 and 6000 mg. The juice was consumed 1 to 6 hours prior to testing. Results indicated that speed of memory was improved with the 750 mg dose but decreased with the highest dose of 6000 mg. Other parameters, including self-reported alertness, followed the same pattern.^123^ Inhaling rosemary oil may have antidepressant effects, potentially leading to significant improvement in vascular health.^124^

###### Ginger

Humans have reported beneficial effects of ginger on pain and inflammation. In a recent human trial, ginger extract produced a moderate reduction in knee pain in 247 patients with osteoarthritis.^125^ The dose of ginger extract that was shown to be effective against knee pain in osteoarthritis and patients with RA was between 0.5 and 1 g daily. Ginger extract may also have antiplatelet, antihypertensive, and hypolipidemic effects.^126^ It has shown the potential to reduce joint swelling, cartilage destruction, circulating levels of inflammatory cytokines associated with RA, and muscle/joint pain.^126^ Bioactive compounds in ginger may also be able to prevent protein glycation and induce thermoregulatory function and fat oxidation in humans, as well as have neuroprotective properties.^126^

A recent meta-analysis concluded that ginger consumption reduced C-reactive protein (CRP) and TNF-α, showcasing its potential to be used for anti-inflammatory complementary herbal adjunct therapy. It may also be effective in decreasing nausea and vomiting during pregnancy.^82^ Antioxidant and anti-inflammatory mechanisms of action of ginger extract include free radical scavenging, inhibition of inflammatory mediator production, and suppression of NF-κB and inflammatory cytokine activity.^82^

## CONCLUSION

In conclusion, herbs and spices clearly contribute to a healthy diet in numerous ways. Their habitual intake may help prevent many cardiometabolic and neurodegenerative diseases, slow down the aging process, and modulate the gut microbiome composition/function. However, despite these benefits, several factors need to be considered when conducting research on herbs and spices and interpreting the results of these studies. For instance, it is important to consider that habitual levels of spice/herb intake are much lower compared with other foods and the significance of their bioactive properties at levels of habitual intake requires careful consideration. As shown by the evidence discussed in this review, many in vitro experiments use doses well above what is consumed in a human diet on a daily basis. The majority of studies conducted at the UCLA Center for Human Nutrition, however, used herbs/spices in their culinary doses, which makes the results more easily translatable to the general population.

It is also relevant to think about how cooking, digestive, and metabolic processes affect beneficial health effects of herbs/spices. As an example, the cooking techniques such as microwaving, simmering, and stewing all increased the antioxidant capacity of cinnamon, cloves, fennel, ginger, parsley, rosemary, sage, and thyme, with heat potentially liberating antioxidant compounds.^55^^,^^81^ On the other hand, evidence shows that dry heating, grilling, and frying may be contributing to a significant decrease in herb/spice antioxidant capacity.^81^ The duration of time spices/herbs are exposed to these various cooking techniques appears to be influential as well. Several experiments demonstrate that cooking time can affect antioxidant capacity and total phenolic content and profile.^55^ Moreover, a recent study showed that digestion significantly increased antioxidant capacity and phenolic content of cooked and uncooked spices.^55^ Another important point to consider is the effect of other foods on the bioavailability of spice/herb bioactive compounds, since herbs/spices are usually consumed as a part of a meal and rarely consumed on their own. This is the reason why the results of dietary intervention trials are challenging to interpret—it is rarely clear which specific food or food compound is driving the observed health effects, as humans usually consume meals and not individual foods or ingredients.

Overall, research on spice/herb effects on human health is a growing field of interest in the research and public health community, especially because spices/herbs are considered natural ingredients and are easily accessible to the general population. Much is already known, but much knowledge/understanding is still lacking. Therefore, more high-quality in vitro experiments, as well as human RCTs, are warranted.

## Data Availability

No new data were created or analysed in this study. Data sharing is not applicable to this article.
